# Ruptured Corpus Luteum among Women Undergoing Laparotomy for Hemoperitoneum in a Tertiary Care Centre: A Descriptive Cross-sectional Study

**DOI:** 10.31729/jnma.7987

**Published:** 2023-02-28

**Authors:** Pooja Paudyal, Suniti Joshi Rawal, Prabhat Khakural

**Affiliations:** 1 Department of Obstetrics and Gynaecology, Tribhuvan University Teaching Hospital, Maharajgunj, Kathmandu, Nepal; 2 Department of Cardiothoracic anaVascular Surgery, Manmohan Cardiothoracic Vascular and Transplant Centre, Maharajgunj, Kathmandu, Nepal

**Keywords:** *anticoagulant*, *corpus luteum*, *hemoperitoneum*

## Abstract

**Introduction:**

Rupture of the corpus luteum, though generally self-limiting in women with normal coagulation, could lead to life-threatening bleeding in patients with prosthetic valves on anticoagulant therapy and described in only a few case reports in the literature. The aim of this study was to find out the prevalence of ruptured corpus luteum among women undergoing laparotomy for hemoperitoneum in a tertiary care centre.

**Methods:**

This descriptive cross-sectional study was conducted among women undergoing laparotomy for hemoperitoneum in a tertiary centre from 7 April 2017 to 31 March 2021 after obtaining ethical approval from the Institutional Review Committee [Reference number: 328(6-11-E)^2^/73/74]. All women who underwent laparotomy for hemoperitoneum during the study period were enrolled. Convenience sampling technique was used. Point estimate and 95% Confidence Interval were calculated.

**Results:**

Out of 447 women who underwent laparotomy for hemoperitoneum, ruptured corpus luteum was seen in 48 (10.74%) (7.87-13.61, 95% Confidence Interval). Out of which 36 (75%) had prosthetic valves. There was 1 (2.77%) mortality and 3 (8.33%) recurrences.

**Conclusions:**

The prevalence of rupture of the corpus luteum among women undergoing laparotomy for hemoperitoneum was similar to other studies done in similar settings. Early diagnosis, emergent reversal of coagulopathy and surgery if needed are the mainstay of management.

## INTRODUCTION

Rupture of the corpus luteum (CL) is a rare complication of ovulation in a woman. A ruptured CL can result in mild to massive hemoperitoneum, which may lead to shock and often emergency surgery.^1,2^ Patients on anticoagulant therapy have a greater risk of extensive hemoperitoneum than those with normal coagulation function.^3,4^

Literature review show only a handful of case reports or case series.^1-5^ As these patients show a higher risk of recurrent bleeding, a study on these women could be helpful in preventing such potentially life-threatening complications.

The aim of this study was to find out the prevalence of ruptured corpus luteum among women undergoing laparotomy for hemoperitoneum in a tertiary care centre.

## METHODS

This descriptive cross-sectional study was conducted in the Department of Obstetrics and Gynaecology.

The duration of the study was from 7 April 2017 to 31 March 2021. Ethical approval was obtained from the Institutional Review Committee of the Institute of Medicine [Reference number: 328(6-11 )^2^/73/74]]. All women who underwent an emergency laparotomy for hemoperitoneum during the study period were enrolled. Women presenting with hemoperitoneum who were managed conservatively without surgery were excluded. A convenience sampling technique was used. The sample size was calculated using the following formula:


$$\begin{array}{ll} \text{n} & ={\text{Z}}^{2}\times \frac{\text{p}\times \text{q}}{{\text{e}}^{\text{2}}}\\ & =1.96^{2}\times \frac{0.50\times 0.50}{0.05^{2}}\\ & =385 \end{array}$$

Where,

n = minimum required sample size
Z = 1.96 at 95% Confidence Interval (CI)
p = Prevalence taken as 50% for maximum sample size calculation
q = 1-p
e = margin of error, 5%

Adding a non-response rate of 10%, the final sample size was 427. However, a total of 447 samples were taken.

Data were collected from patient files regarding age, New York Heart Association (NYHA) class, type and duration of cardiac surgery, and dose of warfarin used. Other variables studied were haemoglobin (Hb gm%) and Prothrombin time International Normalised Ratio (INR) at presentation, the volume of hemoperitoneum, units of blood products transfused and complications.

Data were entered and analysed with IBM SPSS Statistics 24.0. Point estimate and 95% CI were calculated.

## RESULTS

Out of 447 women who underwent laparotomy for hemoperitoneum, the prevalence of patients with ruptured corpus luteum was 48 (10.74%) (7.87-13.61, 95% CI). Out of which 36 (75%) had prosthetic valves and were taking oral anticoagulant therapy. The average age of the patients was 30.44±7.57 years with a range of 18-45 years.

The most common finding was the rupture of the right corpus luteum cyst 34 (70.83%). A total of 12 (25%) had a rupture of left and 2 (4.16%) had a rupture of bilateral corpus luteum cysts.

A total of 36 (75%) women with prosthetic valves underwent surgeries, the most common procedure being mitral valve replacement, 16 (44.44%). All 36 (75%) women were taking the daily oral anticoagulant drug warfarin with an average daily dose of 4.97 mg following the valve replacement procedure (Table 1).

**Table 1:** Types of cardiac surgery (n= 36).

Types of Cardiac Surgery	n (%)
MVR (Mitral valve replacement)	16 (44.44)
AVR (Aortic valve replacement)	3 (8.33)
DVR (Double valve replacement)	10 (27.77)
MVR + tricuspid valve repair	7 (19.44)

The mean duration following surgery was 5.75±4.07 years varying from a minimum of one year to a maximum of 18 years. The majority of 25 (69.44%) of the patients were in New York Heart Association (NYHA) class I and only 1 (2.77%) patient were in NYHA class III (Table 2).

**Table 2:** NYHA class at presentation (n= 36).

NYHA class	n (%)
II	25 (69.44)
II	10 (27.77)
III	1 (2.77)
IV	-

Patients presented to us mainly with complaints of pain abdomen and abdominal distension with an average lag of 2.94 days from onset of symptoms. The average INR on presentation was 4.26±1.26. These patients were managed with a transfusion of 2.8±1.32 units of Packed cells and 3.75±0.96 units of Fresh Frozen Plasma (Table 3).

**Table 3:** Patients characteristics at presentation (n= 36).

Parameters	Mean±SD
Duration of symptoms (days)	2.94±1.63
Warfarin dose (mg)	4.97±1.49
Hb(gm/dl)	7.34±1.93
INR	4.26±1.26
Hemoperitoneum volume (ml)	1179.16±684.35

On laparotomy the average volume of hemoperitoneum was 1179.16 ml and the average duration of hospital stay was 9.75±3.72 days and most of them needed admission to the high dependency unit for a mean duration of 1.75±1.02 days. The most common complication in the postoperative period was arrhythmia in 8 (22.22%) and there was 1 (2.77%) mortality. A total of 3 (8.33%) patients had had a prior episode of hemoperitoneum due to a ruptured corpus luteum cyst and this was their second surgery for the same (Table 4).

**Table 4:** Complications among patients with ruptuted corpus luteum (n= 36).

Parameters	n (%)
Arrhythmia	8 (22.22)
Inotrope requirement	3 (8.33)
Wound infection	1 (2.77)
Pneumonia	1 (2.77)
Acute kidney injury	1 (2.77)
Bowel Injury	2 (5.55)
Seizure	1 (2.77)
Mortality	1 (2.77)

## DISCUSSION

Corpus luteum is a physiological structure which develops from the graffian follicle after ovulation. In the luteal phase of the menstrual cycle, blood vessels penetrating into the wall of the corpus luteum lead to haemorrhage within its cavity, which is usually self-limiting.^1^ Uncommonly it can enlarge in size and rupture as it has a thin wall and is vascular, resulting in hemoperitoneum.^3^ Hemoperitoneum consequent to corpus luteum rupture in a woman has been known to occur but the actual incidence is unknown as it is usually asymptomatic.^6^

Though there are many case series on corpus luteal bleeding reported in the literature^2-5^ corpus luteal bleeding related to prosthetic heart valves has been discussed only in a few case reports.^6-13^ Patients with prosthetic heart valves require lifelong anticoagulation to prevent thromboembolic events however this prolonged anticoagulation puts them at risk for significant and sometimes life-threatening bleeding.^7-9^ Ovarian haemorrhage due to rupture of follicle or corpus luteum requiring emergency interventions has been reported.^6,10^ Studies have shown that treatment with oral anticoagulants increases the risk of major bleeding by 0.3-0.5% per year compared to controls due to a narrow therapeutic index of warfarin, an unpredictable biological response, multiple interactions with concomitant drugs/food and other patient-related factors.^11-13^ This warrants regular monitoring of INR in patients on long-term oral anticoagulants.^7^

In a woman who is undergoing anticoagulant therapy this otherwise self-limiting bleeding could be catastrophic.^1,6,14^ Our study is one of its kind wherein 36 cases of corpus luteal haemorrhage in patients taking warfarin following cardiac surgery were studied. Our patients had been on an anticoagulant for an average of 5.75 years and the haemorrhage was a complication of chronic anticoagulation. According to studies, corpus luteal haemorrhage is frequently a complication when International Normalized Ratio (INR) is more than 4 even though bleeding has been known to occur even when INR is still within or below the therapeutic range.8 In our study average INR was 4.26 (range 1.2-7.5) with an average daily dose of warfarin of 4.97 mg. Regular monitoring of the INR is paramount in patients taking long-term anticoagulation which many of our patients had failed to adhere to. According to one of the studies, rupture can occur at any age between menarche and menopause but is most commonly seen in the third decade of life.^14^ In a study, the mean age was 30 years in our study average age was 30.44 years with the youngest one being 18 and the oldest patient 45 years.^9^ It is reported to occur more on the right ovary as the recto-sigmoid colon is believed to protect the left ovary from trauma.^1,7,14^ Our study also corroborates this finding wherein 25 (69.44%), the rupture was in the right ovary.

Patients of corpus luteal haemorrhage usually present with complaints of sudden abdominal pain due to peritoneal irritation by the blood effusion.^1,7^ Similarly in our study the commonest presenting complaints were abdominal pain and distension. A standard algorithm for the management of such patients is lacking.^4,7^ A high index of suspicion aided with detailed history, clinical examination, lab investigations, pregnancy test and ultrasonography could play crucial roles in accurate diagnosis. Treatment is aimed at reversing the anticoagulation emergently, eliminating the source of bleeding and preserving the ovaries.^7,10,11^ In the present study once the diagnosis was made warfarin was stopped and fresh frozen plasma (FFP) transfused to correct coagulopathy patients were then taken for surgery with the aim to secure hemostasis and attempt to preserve the ovaries as much as possible. Other studies have described a conservative approach with stopping warfarin, replacing clotting factors with FFP and keeping patients under close monitoring when patients presented fairly early with hemoperitoneum less than 1000 ml.^7,8^ With these measures the bleeding stopped often and surgery was reserved for cases with circulatory collapse when other causes of the acute abdomen were suspected or the patient failed to settle on conservative management.^8^ Our cases mostly presented with a lag of 2.94±1.63 days from the onset of symptoms with an average hemoperitoneum of 1179.16 ml, hence surgery was preferred.

The overall estimated mortality in this group of patients has been reported as 3-11%.^5,8,12^ We had one mortality (2.77%) due to refractory shock in a woman with a massive hemoperitoneum of three litres who had presented late and was in shock and atrial fibrillation with NYHA class III at presentation. There were three cases (8.33%) with recurrent episodes of hemoperitoneum. A recurrence rate of 25-31% has been reported in the literature.^5,6^ A suitable method of ovulation suppression is desirable in these patients to prevent recurrences. Combined oral contraceptives are not recommended in these anticoagulated patients. There is good evidence for prescribing depot medroxyprogesterone (DMPA) to suppress ovulation in anticoagulated patients with prosthetic heart valves.^4,9^ In a prospective study of 13 patients has reported the use of depot medroxyprogesterone (DMPA) to suppress ovulation in anticoagulated patients with prosthetic heart valves.^15^ The progestin implants are similar to DMPA in inhibiting ovulation and Gonadotropin Releasing Hormone analogues (GnRHa) are another type of ovulation inhibitor some authors used GnRHa in a patient who had undergone previous surgeries for corpus luteum haemorrhage.^6^ The limitations of the study were the small sample size and single centre. The study did not compare conservative and surgical management as all cases underwent surgery in our centre.

## CONCLUSIONS

The prevalence of rupture of the corpus luteum among women undergoing laparotomy for hemoperitoneum was similar to other studies done in similar settings. Hemoperitoneum resulting from rupture of corpus luteum in patients on oral anticoagulants following cardiac valve replacement surgeries is potentially life-threatening if not diagnosed and treated emergently. Conservative management with emergency reversal of anticoagulation is an option in hemodynamically stable patients and surgical intervention must be resorted to without delay in unstable cases or in presence of a diagnostic dilemmas.
